# Behavioral arrest and a characteristic slow waveform are hallmark responses to selective 5-HT_2A_ receptor activation

**DOI:** 10.1038/s41598-021-81552-6

**Published:** 2021-01-21

**Authors:** April Contreras, Matthew Khumnark, Rochelle M. Hines, Dustin J. Hines

**Affiliations:** University of Nevada, Las Vegas, 4505 Maryland Parkway, Las Vegas, NV 89154 USA

**Keywords:** Neuroscience, Cellular neuroscience, Neural circuits, Neuronal physiology, Synaptic transmission

## Abstract

Perception, emotion, and mood are powerfully modulated by serotonin receptor (5-HTR) agonists including hallucinogens. The 5-HT_2A_R subtype has been shown to be central to hallucinogen action, yet the precise mechanisms mediating the response to 5-HT_2A_R activation remain unclear. Hallucinogens induce the head twitch response (HTR) in rodents, which is the most commonly used behavioral readout of hallucinogen pharmacology. While the HTR provides a key behavioral signature, less is known about the meso level changes that are induced by 5-HT_2A_R activation. In response to administration of the potent and highly selective 5-HT_2A_R agonist 25I-NBOH in mice, we observe a disorganization of behavior which includes frequent episodes of behavioral arrest that consistently precede the HTR by a precise interval. By combining behavioral analysis with electroencephalogram (EEG) recordings we describe a characteristic pattern composed of two distinctive EEG waveforms, Phase 1 and Phase 2, that map onto behavioral arrest and the HTR respectively, with the same temporal separation. Phase 1, which underlies behavioral arrest, is a 3.5–4.5 Hz waveform, while Phase 2 is slower at 2.5–3.2 Hz. Nicotine pretreatment, considered an integral component of ritualistic hallucinogen practices, attenuates 25I-NBOH induced HTR and Phase 2 waveforms, yet increases behavioral arrest and Phase 1 waveforms. Our results suggest that in addition to the HTR, behavioral arrest and characteristic meso level slow waveforms are key hallmarks of the response to 5-HT_2A_R activation. Increased understanding of the response to serotonergic hallucinogens may provide mechanistic insights into perception and hallucinations, as well as regulation of mood.

## Introduction

Serotonin is both a hormone and neuromodulatory neurotransmitter that is involved in executive function, emotions and perceptions. In the nervous system, serotonin is produced by and released from select populations of cells located in the raphe nuclei, which in turn innervate the vast majority of the brain and project extensively to the cortex and thalamus^1–4^. Serotonin acts through binding to receptors (5-HTRs), which include 14 identified family members, divided into 7 subfamilies according to structural and functional properties. Serotonergic hallucinogens act as agonists at 5-HTRs, and are known for their mind-altering qualities, which are best described as alterations in mood, perception, and other cognitive faculties^5–7^. The mind-altering qualities make hallucinogens an attractive agent for a variety of purposes, including a long history of ritualistic practices, and more recently as rapidly acting treatments for disorders like depression and addiction^8–13^.

Although indolamine hallucinogens display activity at a number of receptors^14^, the 5-HT_2A_R is considered central to the mechanism of action of classical hallucinogens^15–18^. The subjective effects of either LSD or ayahuasca in human subjects are blocked by administration of the 5-HT_2A_R antagonist ketanserin^19,20^. In rodents, the head-twitch response (HTR) is a well characterized behavioral readout of hallucinogen pharmacology^15,21–25^. Depending on the drug and dosage used, the rapid head movement is observed as early as 5 min after injection, and can be observed for at least 2 h, with a peak in HTRs around 10 to 20 min following administration^23,26–29^. Administration of 5-HT_2A_R antagonist ketanserin before either LSD or psilocybin blocks the HTR^15,30,31^, and 5-HT_2A_R knockout mice to not display the behavior^15,32^. Also, hallucinogens with higher affinity for the 5-HT_2A_R elicit more HTRs^23^. While the HTR is highly informative about the hallucinogenic potential of a compound, it alone does not provide mechanistic and meso level understanding of the effect of 5-HT_2A_R activation.

Studies using EEG recordings in human subjects have primarily indicated relatively broad suppression a result of hallucinogen action^33,34^, with suppression of α frequency (8–14 Hz) power being the most consistent hallmark^35,36^. Activity in the α frequency band is thought to be reflective of coordinated activity of thalamic pacemaker cells^37^, and to play a role in attention and consciousness^38–40^. More recently, a rise in low frequency bands has been shown to correlate with the peak in subjective experience following hallucinogen use^36,41^. Low frequency band activity is characteristic of sleep, with high amplitude δ (0.5–4 Hz) being characteristic of slow wave sleep, and θ (4–8 Hz) characteristic of REM or subconscious states. It remains unclear what this rise in low frequency bands following activation of 5-HT_2A_R reflects at the meso level.

In Mesoamerican cultures, hallucinogens have been extensively used in ritualistic and religious practices since pre-Columbian times^42^. This long history of use resulted in the accumulation of knowledge about the effects of hallucinogens, and skill in their use^43^. In many of the Mesoamerican hallucinogen practices, wild tobacco (*Nicotiana rustica*) is a common addition^44^. The especially high nicotine content of wild tobacco is thought to enhance the effects of hallucinogens, particularly with respect to the entheogenic effects^44^. Despite this long history of combined use, relatively little research has examined hallucinogen activation of 5-HT_2A_Rs in conjunction with nicotine’s action on nicotinic acetylcholine receptors.

In the present study, we pair detailed behavioral analysis with meso level analysis of cortical EEG in mice to characterize the response to 5-HT_2A_R activation. Using 25I-NBOH, which has both high affinity and specificity for the 5-HT_2A_R, we readily detect the HTR, which is preceded by stops or pronounced episodes of behavioral arrest. Behavioral arrest and the HTR are consistently paired with a relatively short and consistent interval. Using EEG recording, we find an increase in δ and θ frequency band power, and identify two characteristic waveforms that map onto the HTR (Phase 2; P2) and behavioral arrest (Phase 1; P1). P1 is characterized by a waveform of 2–4.5 Hz with consistent EMG signal, while P2 is characterized by waveform of 1–2.5 Hz with sharply rising EMG. We also demonstrate that pretreatment with nicotine strikingly attenuates both the HTR and P2, while increasing the duration of episodes of behavioral arrest as well as the occurrence of P1 waveforms.

## Results

### The 5-HT_2A_R agonist 25I-NBOH leads to disorganized behavior marked by frequent stops

**Figure 1:**
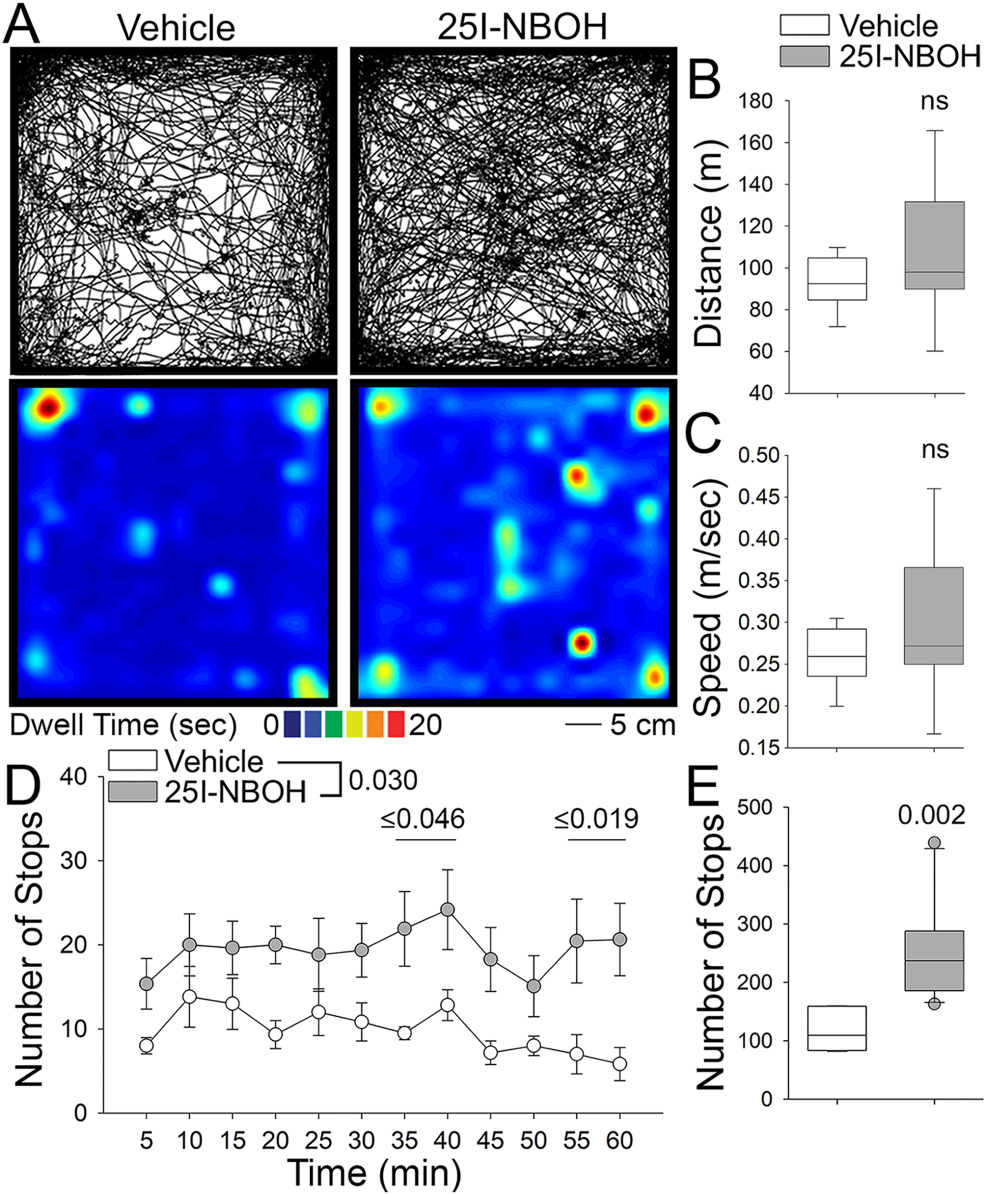
Locomotion in the open field is marked by frequent stops after intraperitoneal injection of 25I-NBOH. (**A**) Representative paths and heat maps for animals treated with vehicle (0.5% DMSO in PBS, left) and 1.429 µg kg^−1^ 25I-NBOH (right) in the open field. (**B**) Box plot comparing total distance traveled following vehicle or 25I-NBOH (ns, not significant). (**C**) Box plot comparing total average speed in the open field (ns). (**D**) A plot of the number of stops made during the 60 min testing period by vehicle and 25I-NBOH treated animals. (**E**) Box plot comparing the number of stops made in the open field by vehicle and 25I-NBOH treated animals. Details of the experimental design and statistical analyses, including numbers of animals, mean ± SE, main effects and p values, can be found in Sup. Table 1.

The indoleamines have been reported to have mixed effects on locomotor behavior, with studies showing both increases and decreases in ambulation in the open field^45,46^. We introduced mice to the open field test and allowed them to freely explore for 60 min immediately following IP injection of 25I-NBOH or vehicle (0.5% DMSO in PBS) control (Fig. 1A). Analysis of 25I-NBOH treated animals reveals no significant change in total distance traveled or average speed compared to vehicle control animals (Fig. 1B,C; Sup. Fig. 1). Despite no change in total distance and average speed, we observed 25I-NBOH treated animals making pronounced stops as they explored the open field, with the number of stops being consistently greater than vehicle control treated mice throughout the 60 min period (Fig. 1D). Overall, 25I-NBOH treated animals had a greater total number of stops, and a greater total time stopped compared to vehicle control animals (Fig. 1E; Sup. Fig. 1D). This detailed examination demonstrates that selective 5-HT_2A_R activation using 25I-NBOH produces an unusual pattern of stops in the open field.

### 25I-NBOH induces stops characterized by behavioral arrest which precede the head-twitch response by a consistent interval

**Figure 2:**
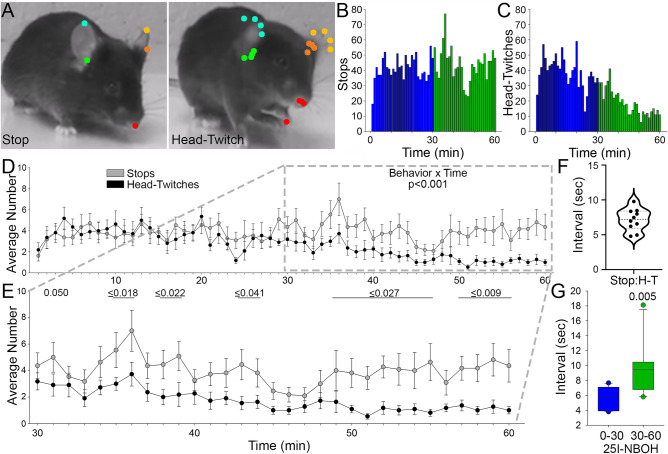
Behavioral arrest consistently precedes the head-twitch response following 25I-NBOH treatment. (**A**) Multi frame composites of a stop and head-twitch with points tracking movement during the distinct behaviors. (**B**) Frequency plot of cumulative stops during the 60 min testing period following 25I-NBOH treatment. (**C**) Frequency plot of cumulative head-twitches during the 60 min testing period following 25I-NBOH treatment. (**D**) Line plot of the average number of stops and head-twitches after 25I-NBOH. (**E**) Line plot highlighting the average number of stops and head-twitches during the last 30 min of the open field. (**F**) Violin plot showing the distribution of the average time interval between a stop and successive head-twitch. (**G**) Box plot comparing the time interval between stop and head twitch pairs during 0–30 and 30–60 min of the open field. Details of the experimental design and statistical analyses, including numbers of animals, mean ± SE, main effects and *p* values, can be found in Sup. Table 1.

Given the high frequency of stops in the open field, we wanted to examine these behavioral episodes in more detail. Using high resolution and high frame rate recording from both above and the side (Fig. 2A) we found that mice began to engage in episodes of behavioral arrest and HTR within the first 10 min of 25I-NBOH (Fig. 2B,C). We found that stops following 25I-NBOH were characterized by an absence of any overt behavior, including visual survey, and also by a high degree of postural control, pointing to a state of behavioral arrest^47^. In contrast, vehicle treated mice routinely engaged in visual survey during stops, and show relaxed posture. In 25I-NBOH treated mice, stops were frequently terminated by an HTR, while in vehicle treated mice stops were most commonly terminated by initiating a new trip in the apparatus, or grooming. HTRs are generally not observed in vehicle treated mice (mean 3.5 ± 0.8) while the incidence of HTRs following 25I-NBOH is notably high (mean 167.5 ± 26.9; Sup. Fig. 3C). Stop frequency was notably high between 5 and 20 min and again between 35 and 50 min following 25I-NBOH (Fig. 2B). Minute to minute examination of the incidence of stops and HTRs showed that the average number of stops and HTRs were nearly identical during the first 20 min of the test (Fig. 2D). Interestingly, this trend changed during the remaining 40 min of the test, with the frequency of the HTR decreasing and the number of stops remaining relatively unchanged (Fig. 2E). Of interest, the peak of subjective experience following hallucinogen use is typically observed after 30 min^48^, although HTRs induced by a range of hallucinogens are known to decline after 30 min^28,49^. We next detected behavioral arrest and HTR pairs in 25I-NBOH treated mice and examined the temporal relationship between the two events. We noted that stops consistently preceded HTRs by relatively short intervals, with HTRs typically following stops within 5 to 10 s (mean = 7.652 ± 0.532 s; Fig. 2F,G). These findings demonstrate that 5-HT_2A_R activation using 25I-NBOH produces marked episodes of behavioral arrest, which temporally precede the characteristic HTR.

**Figure 3:**
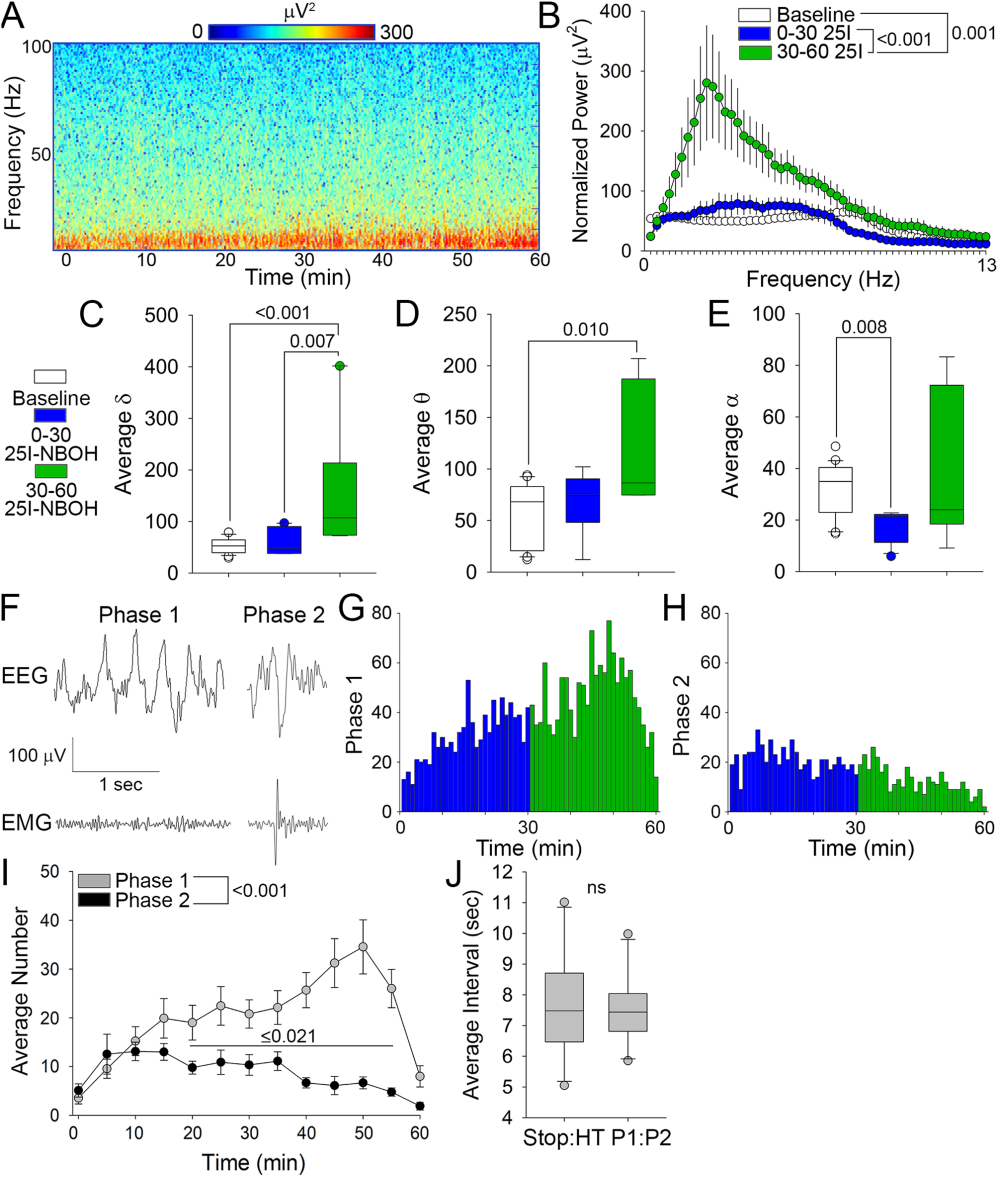
25I-NBOH produces a characteristic two waveform complex that drives increased low frequency power. (**A**) Spectrogram of representative EEG recordings after administration of 25I-NBOH. (**B**) FFT of EEG recordings comparing baseline, 0–30 min, and 30–60 min after 25I-NBOH. (**C**–**E**) Box plot of normalized power from EEG recordings parsed into δ (**C** 0.4–4.0 Hz), θ (**D** 4.5–8 Hz), and α (**E** 8.5–13 Hz) frequency bands. (**F**) Representative Phase 1 and Phase 2 waveforms occurring in the EEG and EMG from epoch analysis. (**G**) Plot of the cumulative Phase 1 waveforms occurring in the EEG after administration of 25I-NBOH. (**H**) Plot of the cumulative Phase 2 waveforms occurring in the EEG after administration of 25I-NBOH. (**I**) Average number of Phase 1 and Phase 2 waveforms. (**J**) Box plot comparing the time interval between a stop and head-twitch observed in the open field and the interval between Phase 1 and Phase 2 waveforms in the EEG recordings (ns). Details of the experimental design and statistical analyses, including numbers of animals, mean ± SE, main effects and p values, can be found in Sup. Table 1.

### A two-waveform complex underlies the behavioral response to 25I-NBOH

To examine the meso level waveforms that underly the behavioral arrest and HTR induced by 5-HT_2A_R activation, we conducted EEG recording paired with behavioral assessment in mice treated with 25I-NBOH. For these studies, mice were examined for a 60-min period post 25I-NBOH injection, and compared to their own pre-injection baseline. Spectrograms revealed an increase in power of slow waveforms, particularly 30–60 min post 25I-NBOH injection (Fig. 3A). To resolve the underlying frequencies of the EEG that were affected, we performed fast Fourier transform (FFT) analysis and compared baseline activity to that recorded between 0–30- and 30–60-min post 25I-NBOH. This analysis confirmed an increase in the power of low frequency bands, specific to the 30–60-min period (Fig. 3B; Sup. Fig. 2A). We also performed spectral analysis and found that both δ and θ frequency band power were significantly increased 30–60 min post injection (Fig. 3C,D), while α frequency band power was suppressed between 0–30 min following 25I-NBOH (Fig. 3E). Change was not observed in either the β or γ frequency band (Sup. Fig. 2A inset, B,C). Given the striking and relatively selective increase in both δ and θ frequency band power 30–60 min following 25I-NBOH treatment, we were interested to examine the source of this power change.

To examine the waveforms driving the increase in δ and θ frequency band power, we parsed the EEG and EMG traces into 10 s epochs to manually examine waveform activity post 25I-NBOH injection. Epoch analysis revealed two distinct yet characteristic waveforms occurring in the EEG. The first waveform (Phase 1; P1) is a distinctive 3.5–4.5 Hz waveform with consistent low EMG signal (Fig. 3F), which upon analysis of synchronized video is associated with behavioral arrest. The second waveform (Phase 2; P2) is a 2.5–3.2 Hz waveform with initial low but sharply rising power in the EMG signal (Fig. 3F), which mapped onto the HTR in synchronized video. Each waveform lasted around 1 s. P1 and P2 waveforms are distinctive from normal EEG waveform activity, and were not observed in baseline recordings. To examine the relationship between the waveforms and the behavioral response to 5-HT_2A_R activation, the number of P1 and P2 waveforms in the EEG trace were plotted for the time post 25I-NBOH injection (Fig. 3G–I). In support of their relationship, the frequency of P1 and P2 waveforms followed a similar temporal pattern to the frequency of stops and HTRs in the open field. While the P1 counts continued to increase (Fig. 3G,I), the P2 counts were reduced during the last 30 min (Fig. 3H,I). Also, in support of the relationship between the behavioral and meso level effects of 25I-NBOH, P1 preceded P2 by a consistent interval of 5 to 10 s (mean = 7.521 ± 0.365 s), which did not significantly differ from the stop to HTR interval (7.652 ± 0.532 s; Fig. 3J). The high prevalence of P1, with a frequency of 3.5–4.5 Hz, likely contributes to the elevation in δ and θ frequency band power detected 30–60 min following 25I-NBOH. These findings demonstrate that a two-waveform complex underlies the behavioral arrest and HTR observed following 25I-NBOH activation of 5-HT_2A_Rs.

### Pretreatment with nicotine attenuates the head-twitch response but enhances behavioral arrest post 25I-NBOH

**Figure 4:**
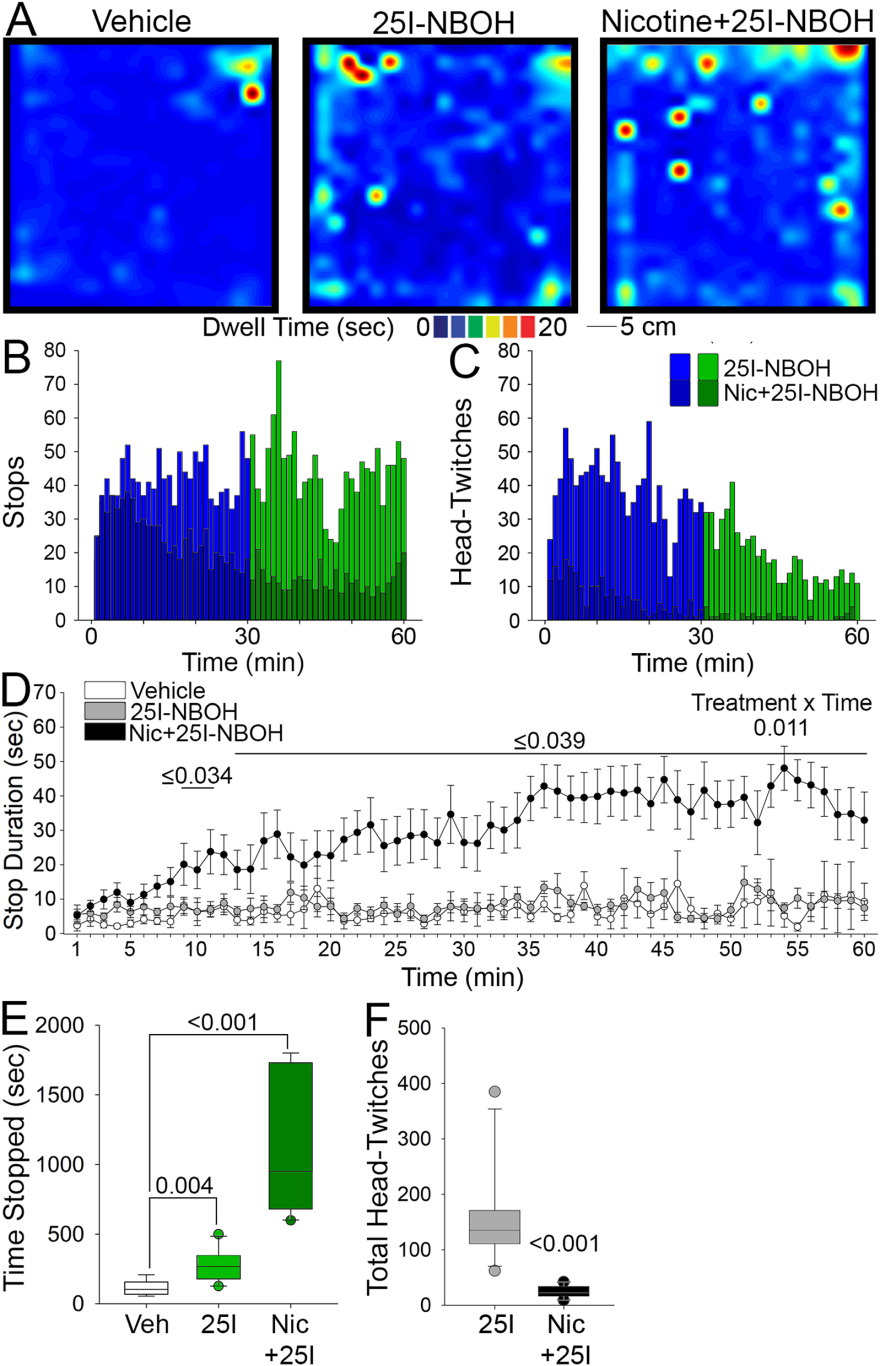
Behavioral arrest in the open field is increased after pretreatment with nicotine. (**A**) Representative heat maps for animals treated with vehicle (left), 1.429 µg kg^−1^ 25I-NBOH (middle), and 2 mg kg^−1^ nicotine in the open field. (**B**) Plot of the cumulative stops made in the open field by nicotine pretreated animals (superimposed dark blue and green) compared to 25I-NBOH treatment alone (bright blue and green). (**C**) Plot of the cumulative head-twitches made by nicotine pretreated animals (superimposed dark blue and green) compared to 25I-NBOH treatment alone (bright blue and green). (**D**) Line plot of duration of stops made during the 60 min testing period, comparing vehicle, 25I-NBOH and nicotine + 25I-NBOH treated animals. (**E**) Comparison of the time stopped in the open field for vehicle, 25I-NBOH, and nicotine + 25I-NBOH treated animals during the 30–60 min time period. (**F**) Total number of head-twitch for 25I-NBOH and nicotine pretreated animals. Details of the experimental design and statistical analyses, including numbers of animals, mean ± SE, main effects and p values, can be found in Sup. Table 1.

The incorporation of tobacco into Mesoamerican hallucinogen rituals has been well-documented and nicotine from tobacco is considered a key element in these practices^42,44^. To examine the influence of nicotine on behavioral arrest and the HTR, animals were given an oral dose of nicotine and returned to their home cage for 30 min before injection with 25I-NBOH and being placed in the open field. 25I-NBOH combined with nicotine pretreatment resulted in both behavioral arrest and the HTR (Fig. 4A–C). Although both behavioral arrest and HTR episodes occur with nicotine pretreatment, they are much less frequent than 25I-NBOH alone. While the frequency of stops following 25I-NBOH was reduced by nicotine pretreatment, we noticed that the stops were of significantly longer duration. Plotting stop duration over time shows that nicotine pretreatment increases stop duration above 25-INBOH alone (and vehicle control) within the first 30 min after treatment (Fig. 4D). We next compared the cumulative stop duration and found that nicotine pretreatment increased the duration stopped in the open field, during both 0–30 and 30–60 min following 25I-NBOH (Fig. 4E). In contrast to behavioral arrest, the HTR was nearly abolished by nicotine pretreatment (Fig. 4F). This is consistent with a prior study demonstrating that pretreatment with nicotine attenuated the HTR after dosing of (+/−)-1-(2,5-dimethoxy-4-iodophenyl)-2-aminopropane (DOI)^50^. Nicotine treatment alone did not increase the time stopped in the open field (Sup. Fig. 3A,D) or elicit notable HTRs (Sup. Fig. 3C,D), and was similar to vehicle. These findings demonstrate that pretreatment with nicotine substantially alters the behavioral response to 25I-NBOH by sharply reducing the HTR, yet increasing the duration of behavioral arrest episodes.

### Pretreatment with nicotine increases Phase I waveforms following 25I-NBOH

**Figure 5:**
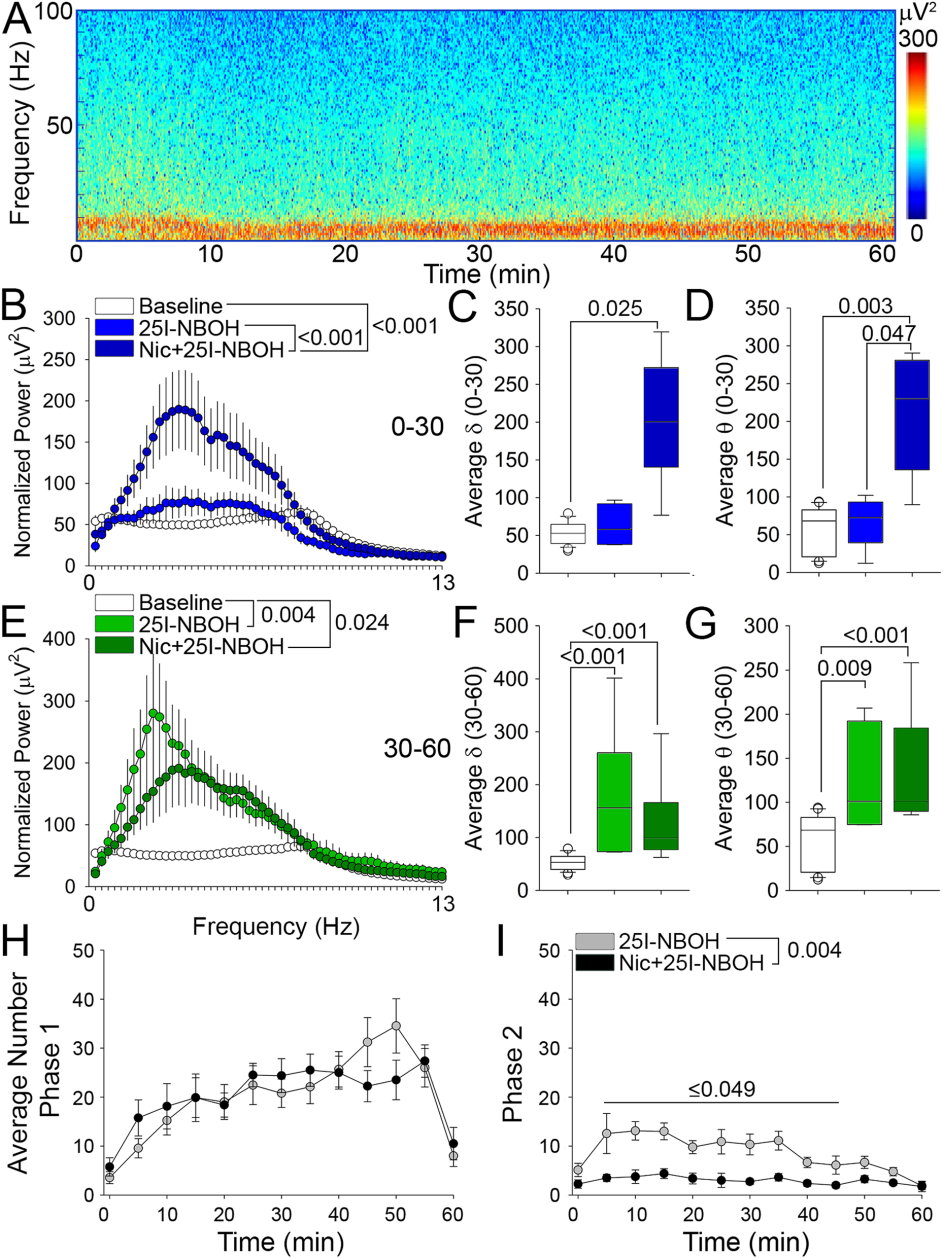
EEG analysis indicates pretreatment with nicotine increases Phase 1 waveforms. (**A**) Spectrogram of representative EEG recordings after 25I-NBOH injection for animals pretreated with nicotine. (**B**) FFT of EEG recordings comparing baseline, 25I-NBOH, and nicotine + 25I-NBOH treated animals during 0–30 min of EEG recordings. (**C**,**D**) Box plot of normalized power from EEG recordings parsed into δ (**C** 0.4–4.0 Hz) and θ (**D** 4.5–8 Hz) frequency bands comparing vehicle, 25I-NBOH, and nicotine + 25I-NBOH treated animals during 0–30 min. (**E**) FFT of EEG recordings comparing baseline, 25I-NBOH, and nicotine + 25I-NBOH treated animals during 30–60 min of EEG recordings. (**F**,**G**) Box plot of normalized power from EEG recordings parsed into δ (**F** 0.4–4.0 Hz) and θ (**G** 4.5–8 Hz) frequency bands comparing vehicle, 25I-NBOH, and nicotine + 25I-NBOH treated animals during 30–60 min. (**H**) Line plot comparing the average number of Phase 1 waveforms observed in 25I-NBOH and nicotine + 25I-NBOH treated animals. **I** Line plot comparing the average number of Phase 2 waveforms observed in 25I-NBOH and nicotine + 25I-NBOH treated animals. Details of the experimental design and statistical analyses, including numbers of animals, mean ± SE, main effects and p values, can be found in Sup. Table 1.

Because nicotine pretreatment increased the duration of behavioral arrest episodes and strongly attenuated the HTR following 25I-NBOH, we next examined how nicotine influenced P1 and P2 waveform complexes by pretreating animals with nicotine after 60-min of baseline recording. To mirror the behavioral studies, nicotine pretreatment was followed 30 min later by 25I-NBOH injection. Representative spectrograms showed increased power in the low frequencies with nicotine pretreatment, beginning within the first 30 min following 25I-NBOH (Fig. 5A). Nicotine pretreatment elevated low frequency power during both 0–30- and 30–60-min intervals following 25I-NBOH treatment (Fig. 5B,E; Sup. Fig. 4). Nicotine pretreatment resulted in a significant increase in both δ and θ frequency band power during the first 30 min following 25I-NBOH, which was not observed with 25I-NBOH alone (Fig. 5C,D; Sup. Fig. 4). Low frequency band power spanning both δ and θ was enhanced 30–60 min following 25I-NBOH with nicotine pretreatment, similar to 25I-NBOH alone (Fig. 5F,G; Sup. Fig. 5). α power is suppressed by 25I-NBOH alone during the 0–30 min period, but not by 25I-NBOH with nicotine pretreatment (Sup. Fig. 5B,F). Epoch analysis showed that P1 waveforms with nicotine pretreatment were similar in frequency to 25I-NBOH alone, and followed a similar temporal pattern (Fig. 5H). However, P2 waveforms were significantly less frequent with nicotine pretreatment compared to 25I-NBOH alone (Fig. 5I). These findings demonstrate that nicotine pretreatment attenuates P2 waveform frequency in parallel with the decrease in the HTR. Despite this attenuation of P2, P1 is maintained with nicotine pretreatment, consistent with long duration behavioral arrest observed with combined nicotine and 25I-NBOH. These data demonstrate that behavioral arrest and the associated low frequency P1 waveform are consistent hallmarks of 5-HT_2A_R activation, which are enhanced by nicotine pretreatment.

## Discussion

We demonstrate that the highly potent and specific 5-HT_2A_R agonist 25I-NBOH induces a disorganization of behavior marked by behavioral arrest and a robust HTR. Despite no overall change in distance traveled or average speed, our results show that 25I-NBOH activation of 5-HT_2A_Rs affects the number of stops made in the open field, which were characterized by an absence of visual survey or grooming behaviors. These frequent stops closely preceded a robust HTR, and the two behavioral events were separated by a short and precise interval. To understand the waveforms induced by 5-HT_2A_R activation, we conducted cortical EEG recording and found an increase in δ and θ frequency band power 30–60 min after 25I-NBOH treatment. Meso level analysis revealed a distinct two-waveform complex, which temporally matched with behavioral arrest (P1) and the HTR (P2) observed in video recordings of the mice. P1 is 3.5–4.5 Hz waveform accompanied by consistent EMG, while P2 is slower at 2.5–3.2 Hz and accompanied by sharply rising EMG signal. P1 and P2 were separated by an interval that mirrors the interval between behavioral arrest and the HTR. Pretreatment with nicotine nearly abolishes the HTR and P2 following 25I-NBOH injection, however, the duration of episodes of behavioral arrest increased in parallel with consistent occurrence of P1. Our results indicate that selective 5-HT_2A_R activation induces hallmark responses of behavioral arrest and the HTR, accompanied by characteristic slow waveforms. The occurrence of these slow waveforms, particularly P1, contributes to the increase in δ and θ frequency band power observed following 5-HT_2A_R activation.

Exploratory behavior tasks like the open field have been used to assess the effects of hallucinogens in rodents with varied and inconsistent results, suggesting that locomotion may not enable discrimination of hallucinogenic effects from those of other drugs^14^. In agreement with this, we found that acute IP injection of the specific 5-HT_2A_R agonist 25I-NBOH did not significantly change broad measures of locomotor activity, including total distance travelled or average speed. While assessments of locomotion showed no difference, we did find that 25I-NBOH treated animals made significantly more frequent stops as they traversed the arena compared to vehicle controls. This finding is consistent with a classic report examining LSD in rats, which indicated bouts of sitting motionless^45^, however this behavioral response has not been a focus for further study. We found that stops following 25I-NBOH were characterized by behavioral arrest, and temporally related to the HTR, which is a behavioral hallmark of the response to hallucinogens in animal models^23,26,28^. An extensive body of work has shown that the HTR in rodents is correlated with subjective hallucinogenic experiences reported in human studies^14,15,21–23,25^. We now describe behavioral arrest as a key feature of selective 5-HT_2A_R activation, related to the HTR, which maps temporally onto the peak subjective experience reported in human subjects^48^. Behavioral arrest is observed under a few specific contexts in rodents, including upon reaching a goal, in response to threat, as well as in response to surprising visual and auditory stimuli^47^. Thus, behavioral arrest observed following 5-HT_2A_R activation by 25I-NBOH may be related to unexpected stimuli that are not actually present.

In conjunction with behavioral arrest, we observe a characteristic slow waveform that contributes to elevations in δ and θ frequency band power following 25I-NBOH activation of 5-HT_2A_Rs. This is consistent with recent findings of enhanced δ and θ power that parallels the peak subjective experience in human subjects following hallucinogen administration^36,41^. Elevations in δ and θ power are most consistently associated with sleep or subconscious states in both rodents and humans. Prior studies have associated behavioral arrest with an interruption of awake cortical activity, characterized by an increase in low frequency power^51^. Behavioral arrest and interruption of awake cortical activity can be induced by activation of inhibitory fibers from the pontine reticular formation to the intralaminar thalamus^51^. Of interest, the intralaminar thalamus also receives extensive serotonergic innervation^52^ and is interconnected with the frontal cortex^53^. The intralaminar thalamus is thought to influence higher order cortical functions by modulating the degree of synchrony between cortical areas^54^. This is in keeping with studies demonstrating that hallucinogens may influence cortical activity via activation of thalamic 5-HT_2A_Rs^55–57^.

Hallucinogens have been used by knowledgeable and highly skilled pre-Columbian Mesoamerican practitioners for over 5000 years, with the ritualistic and religious use of hallucinogens often incorporating the use of tobacco (*Nicotiana rustica*)^42^. It is thought that the addition of nicotine enhances the effects of hallucinogens on states of consciousness^44^. Of interest, HTRs induced by 25I-NBOH and other hallucinogens are attenuated by nicotine pretreatment^50^. In contrast, we found that nicotine pretreatment increased the duration of episodes of behavioral arrest, in parallel with enhanced δ and θ frequency band power and the occurrence of P1. In addition to robust serotonergic innervation, the thalamus is also particularly dense with α4β2 nicotinic acetylcholine receptors^58,59^, providing a possible mechanism for the enhancement of hallucinogen action by nicotine, via further modulation of thalamocortical connectivity^60,61^ and enhancement of low frequency band δ and θ power. Of particular interest, enhanced δ and θ power has also been observed during hallucinations in schizophrenic subjects, and in bouts of creativity in typical subjects^62^, suggesting that this pattern of EEG activity during wake may be associated with generation of internal perceptions of a variety of origins.

Collectively, our results suggest that behavioral arrest and a characteristic 3.5–4.5 Hz waveform are important hallmarks of the response to 5-HT_2A_R activation, and may be central to the mechanisms and effects of serotonergic hallucinogens. Further, we show that nicotine enhances behavioral arrest and the associated slow waveform, providing a potential mechanism for nicotine’s enhancement of hallucinogen action. In both ritualistic as well as contemporary clinical applications, hallucinogens acting on 5-HT_2A_Rs have been shown to promote well-being^63^, including in the context of addiction^9,10,13^ and treatment resistant depression^8,11,12^. Thus, the described data may help refine the use of hallucinogens in clinical practice by providing meso-level hallmarks of robust yet selective 5-HT_2A_R activation, advancing treatment of psychiatric disorders.

## Methods

### Animals

Animals were cared for according to the NIH Guide for the Care and Use of Laboratory Animals^64^, and protocols were approved by the Institutional Animal Care and Use Committee (IACUC) of the University of Nevada, Las Vegas. Mice were group housed with a 12 h light-dark cycle with constant access to food and water. One animal was removed from the behavioral study due to a missed injection. The study was designed and carried out in compliance with the ARRIVE guidelines**.**

### Pharmacology

25I-NBOH (Cayman Chemical, Ann Arbor, MI) was prepared in DMSO (0.5%, in PBS) and was administered intraperitoneally according to the weight of the animal to achieve a 1.5 × 10^−5^ mg kg^−1^ dose. The vehicle consisted of 0.5% DMSO in PBS. Nicotine (free base) was administered orally at a dose of 0.2 mg kg^−1^, either alone as an additional control group, or 30 min prior to treatment with 25I-NBOH.

### Behavioral assessments

All behavioral assessments were conducted within the first four hours of the dark cycle, when mice are known to be most active. The open field apparatus was designed based on the European Mouse Phenotyping Resource for Standardized Screens (EMPReSS)^65^. Mice were habituated to the testing room for 1 h before testing and randomized into treatment groups. Open field behavior of mice was assessed using ANY-Maze video tracking software from video recordings taken from above. Mice were either dosed with 25I-NBOH or vehicle control before being placed in the open field for 60 min, or pretreated with nicotine for 30 min before 25I-NBOH injection and being placed in the open field for 60 min. Total distance travelled and average speed of all animals was tracked using ANY-Maze. Heat maps depicting movement and immobility in the open field were created using Matlab.

High resolution recordings of behavior from the side were captured with a GoPro camera. Animals were placed in a Plexiglass chamber for 30 min immediately following 25I-NBOH injection. Using a GoPro, we were able to closely examine stops, the rapid, sequential side-to-side movements of the HTR, as well as other behaviors such as grooming and rearing. The number of stops, duration of stops, and HTRs made by the animals were hand scored, in parallel with kinematic analysis using deep lab cut pro. Measures were cross validated. The HTR was assessed by a trained scorer who was blinded to the treatment via visual examination of open field, EEG, and higher resolution GoPro video recordings. Criteria for a complete HTR included a rapid paroxysmal rotational head movement, with little to no movement in the torso of the animal. HTRs were only counted if they could be clearly observed and distinguished from other behaviors. Because mice can be observed to make head movements during grooming, sniffing, and rearing behaviors, episodes were excluded if the animal only titled its head to one side. Stops were also assessed by a trained scorer. Criteria for a stop included an immobile episode lasting at least 2 s. A stop was considered terminated if the animal moved to a new location in the behavioral apparatus, began any phase of the grooming circuit, or in the case of 25I-NBOH treated mice, if an HTR was observed. The time interval between a stop and an HTR was calculated as the length of time (seconds) between an initiated stop and the display of an HTR.

### Electroencephalography

Electroencephalography (EEG) and electromyography (EMG) electrodes were implanted under isoflurane anesthesia. Two channels of EEG were recorded bilaterally from the frontal cortex, with ground supplied by placement of an electrode in the caudal parietal area^66,67^. Following implantation, mice were divided randomly into 25I-NBOH or 25I-NBOH with nicotine pretreatment (n = 6) groups. EEG data before administration provided baseline, allowing the same animals to be used as their own control. After a minimum of 7 days of postoperative recovery, EEG activity was recorded at a sampling rate of 1000 Hz using the Pinnacle system for mouse during the first four hours of the dark phase of the 24 h cycle^66,67^. Mice were first acclimatized to the recording chamber and preamplifier for 60 min, then injected with 0.5% DMSO in PBS vehicle before recording at least 60 min of baseline. During baseline recordings, sleep was suppressed or interrupted by introduction of novel objects as necessary following procedures from our prior studies^66,67^. After at least 60 min of baseline, mice were administered 25I-NBOH via intraperitoneal injection. Recordings proceeded for at least 60 min following administration. Mice were not observed to sleep following administration based on assessment of behavioral or EEG characteristics by a trained researcher. Results were plotted, analyzed, and quantified using MatLab and SleepSign. MatLab was used to generate spectrograms of the EEG data. SleepSign was used to manually scroll through each recording and quantify instances of P1 and P2 waveforms, as well as generate FFT data sets. Individual FFT data sets were normalized to the average power of the EEG between 0.4 and 100 Hz. Transformed and normalized data were parsed into spectral frequency band divisions, defined as follows: δ-0.4–4 Hz, θ-4–8 Hz, α-8–13 Hz, β-13–30, γ-30–100 Hz^66,67^. Spectral analysis compared 60 min of baseline to 60 min post administration^66,67^. For nicotine pretreatment experiments, spectral analysis compared 60 min of baseline to 60 min post administration of 25I-NBOH (which followed nicotine by 30 min). We also calculated and plotted the log_10_ of γ frequency band values to more readily allow comparison of the very low power fast frequencies.

### Statistics

Graphs were plotted as mean ± standard error (line graphs); minimum, first quartile, median, third quartile, and maximum (box plots); or median, interquartile range, and kernel density plot (violin plot) using SigmaPlot or GraphPad. Provided that the data met assumptions of normality (Shapiro–Wilk) and equal variance (Brown-Forsythe), results were analyzed using ANOVA (one-way, or two-way repeated measures as appropriate) with Bonferroni post hoc. If the data did not meet assumptions of either normality or equal variance, a Kruskal–Wallis one-way ANOVA on ranks was used, with Dunn’s method or Tukey test multiple comparisons applied where appropriate. Where space permits, actual *p* values are shown in the figures, while “ns” is used to denote a non-significant result from statistical analysis of particular data sets. Details of the groups compared, numbers of animals used, the statistical analyses performed, means and standard error, and main effects can be found in Sup. Table 1.

## Supplementary Information

Supplementary Information.
