# Supplementary material for: Behavioral arrest and a characteristic slow waveform are hallmark responses to selective 5-HT2A receptor activation
Source: Sci Rep. 2021 Jan 21;11:1925. doi: 10.1038/s41598-021-81552-6 (PMC7820508; doi:10.1038/s41598-021-81552-6)
Supplement: Supplementary file 1 — Supplementary Information. [file 41598_2021_81552_MOESM1_ESM.pdf]

Behavioral arrest and a characteristic slow waveform are hallmark responses to selective 5-HT_{2A} receptor activation

April Contreras, Matthew Khumnark, Rochelle M. Hines, Dustin J. Hines[1]

University of Nevada, Las Vegas, 4505 Maryland Parkway, Las Vegas, NV, USA, 89154

[1]Corresponding author, email: dustin.hines@unlv.edu; 702-895-2208

Supplementary Information

Supplementary Figures

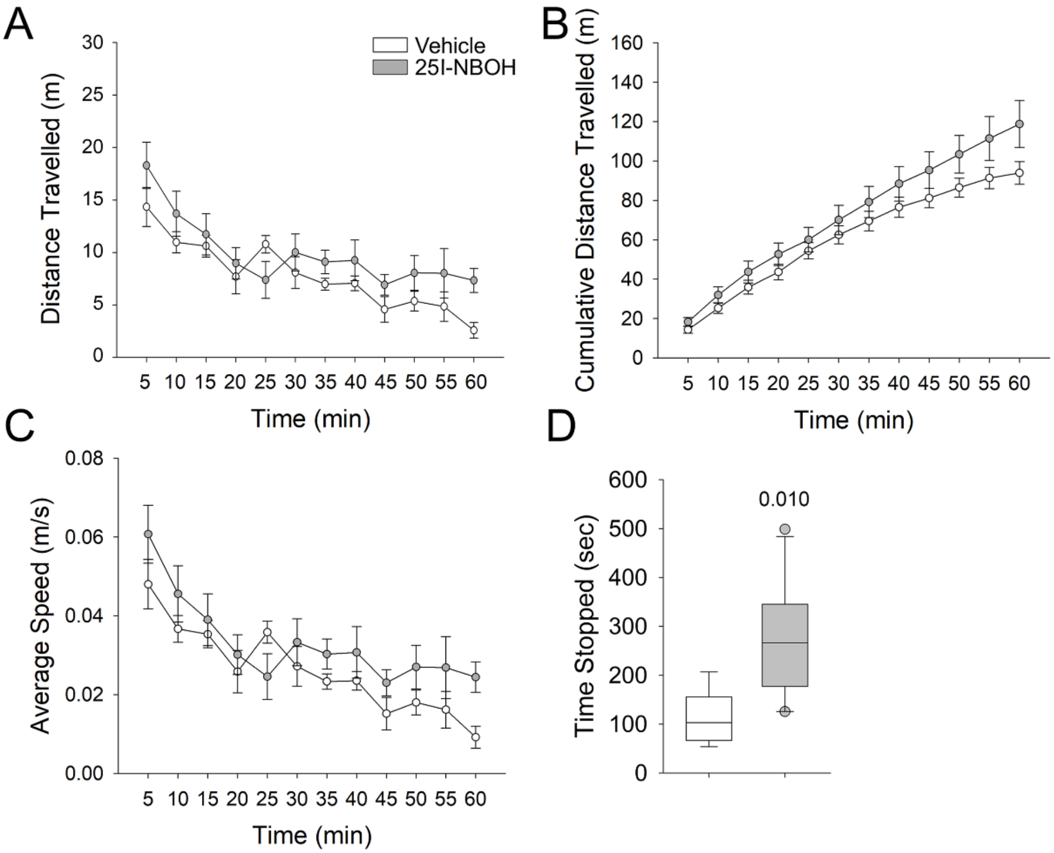

Sup Fig 1: Global assessments of locomotion are not significantly altered by 25I-NBOH while time stopped increases. A. Distance travelled over time, and B. cumulative distance travelled in the open field comparing vehicle and 25I-NBOH treated mice. C. Average speed of trips in the open field comparing vehicle and 25I-NBOH treated mice. D. Time stopped in the open field comparing vehicle and 25I-NBOH treated mice. Details of the experimental design and statistical analyses, including numbers of animals, mean ± SE, main effects and p values, can be found in Sup. Table 1.

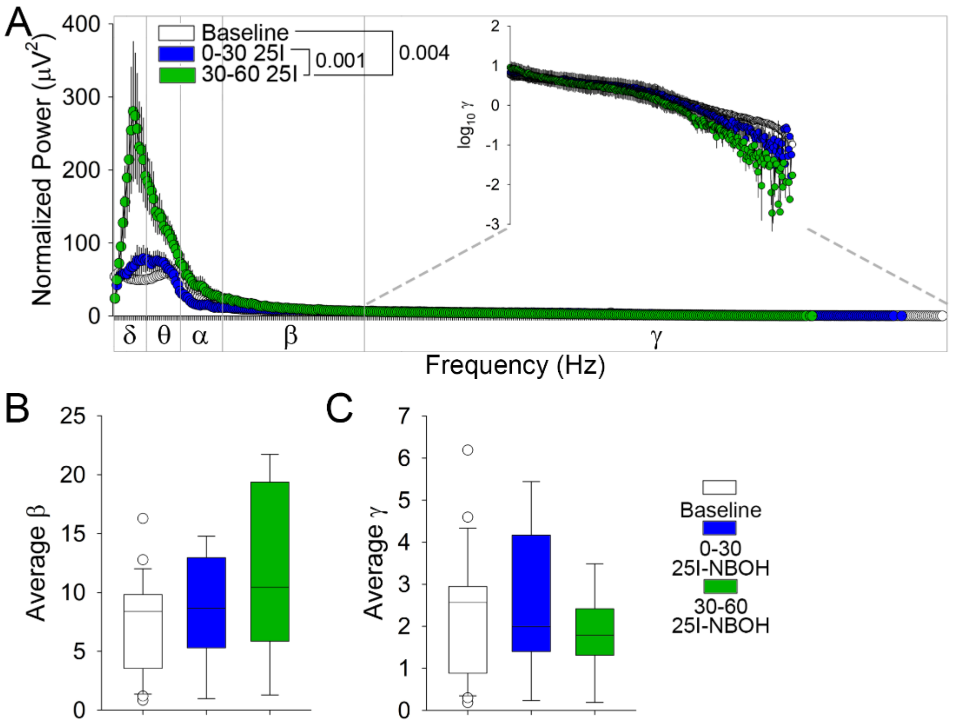

Sup Fig 2: 25I-NBOH increases low frequency (δ and θ) power in the EEG along with a suppression of α. A. FFT for the entire recorded spectrum (0.4-100 Hz). Inset: log_{10} for the high frequency γ band of the EEG. B. β power is unaltered by 25I-NBOH. C. γ power is not significantly impacted by 25I-NBOH. Details of the experimental design and statistical analyses, including numbers of animals, mean ± SE, main effects and p values, can be found in Sup. Table 1.

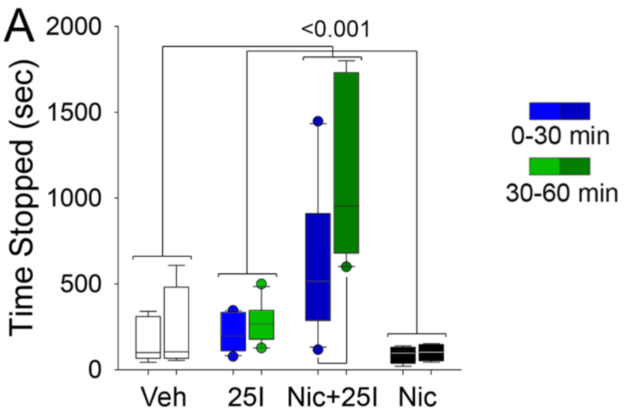

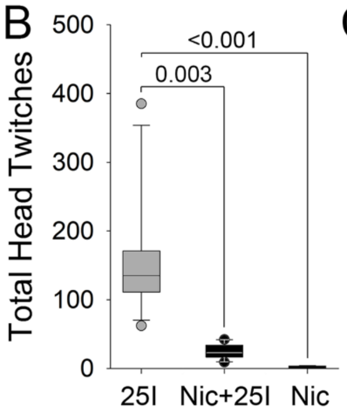

C

Treatment	Stops	Head-Twitches
Vehicle	117.3±14.8	3.5±0.8
25I-NBOH	249.4±34.1	167.5±26.9
Nicotine+25I	111.8±17.6	24.7±3.3
Nicotine	102.5±30.1	1.5±1.0

Sup Fig 3. Modulation of 25I-NBOH induced behavioral arrest and head twitch response by nicotine pretreatment. A. Time stopped comparing vehicle, 25I-NBOH (25I), nicotine pretreatment followed by 25I-NBOH (Nic+25I), and nicotine alone treatment (Nic), during both the 0-30 and 30-60 minute time periods. B. Incidence of the head twitch response comparing 25I-NBOH (25I), nicotine pretreatment followed by 25I-NBOH (Nic+25I), and nicotine alone treatment (Nic). C. Descriptive statistics for the number of stops and the number of head twitches across all groups (mean ± SE). Details of the experimental design and statistical analyses, including numbers of animals, mean ± SE, main effects and p values, can be found in Sup. Table 1.

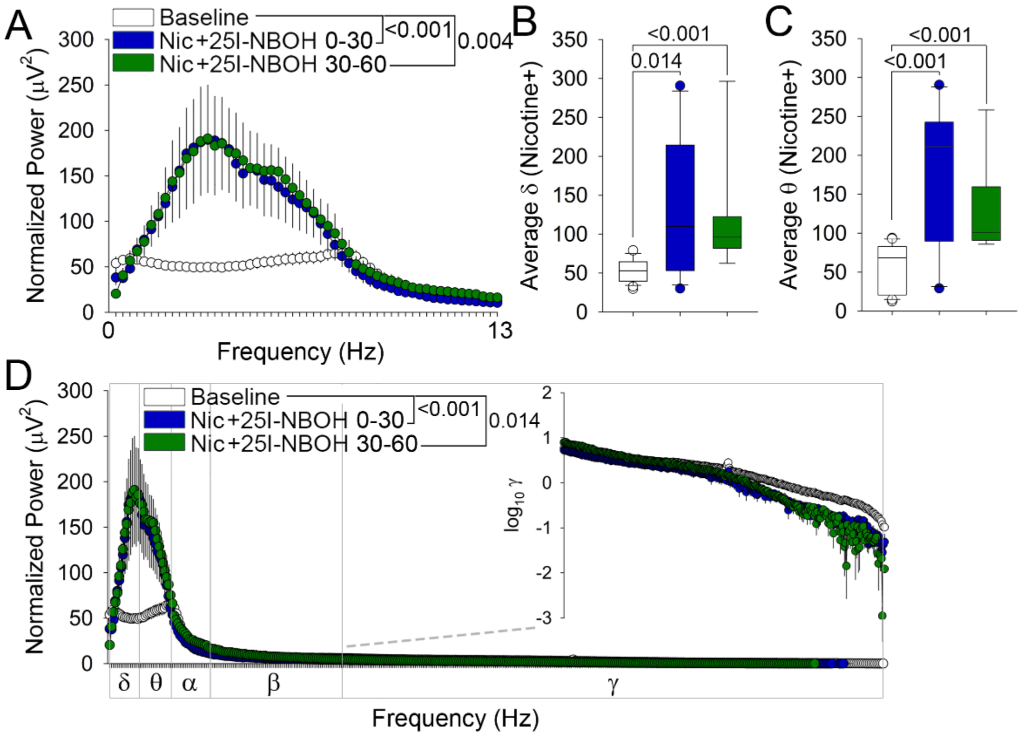

Sup Fig 4: Nicotine pretreatment increases low frequency power both 0-30 and 30-60 minutes following 25I-NBOH. A. FFT for low frequencies (0.4-13 Hz) with nicotine pretreatment followed by 25I-NBOH. Elevation of δ (B.) and θ (C.) frequency band power is observed during the entire 60 minutes with nicotine pretreatment followed by 25I-NBOH. D. FFT for the entire recorded spectrum (0.4-100 Hz) with nicotine pretreatment followed by 25I-NBOH. Inset: $\log_{10}$ for the high frequency γ band of the EEG. Details of the experimental design and statistical analyses, including numbers of animals, mean ± SE, main effects and p values, can be found in Sup. Table 1.

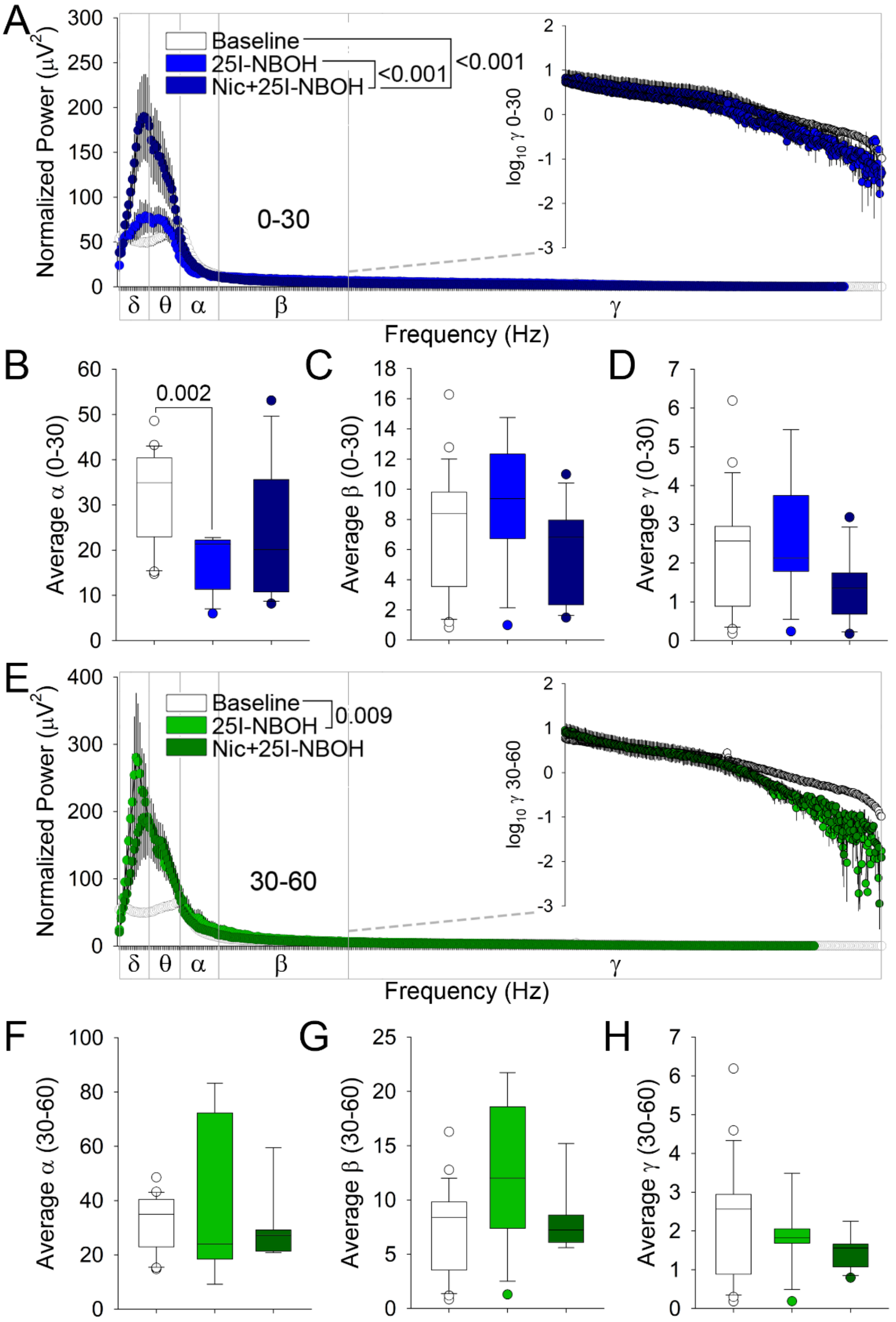

Sup Fig 5: Nicotine pretreatment enhances the effects of 25I-NBOH on low frequency power. A. FFT for the entire recorded spectrum (0.4-100 Hz) 0-30 minutes following treatment with 25I-NBOH, and nicotine pretreatment followed by 25I-NBOH compared to baseline. Inset: log_{10} for the high frequency γ band of the EEG. B. Suppression of α power is observed only with 25I-NBOH 0-30 minutes following treatment. C. No change in β power. D. No change in γ power. E. FFT for the entire recorded spectrum (0.4-100 Hz) 30-60 minutes following treatment with 25I-NBOH, and nicotine pretreatment followed by 25I-NBOH compared to baseline. Inset: log_{10} for the high frequency γ band of the EEG. F-H. No change in α, β, or γ power 30-60 minutes following 25I-NBOH or nicotine plus 25I-NBOH. Details of the experimental design and statistical analyses, including numbers of animals, mean ± SE, main effects and p values, can be found in Sup. Table 1.

Sup. Table 1. Summary of study design, sample size (n=individual animals), and statistical analyses performed.

Fig.	Panel	Groups (n)	Test<br>Post Hoc	Mean ± SE<br>or LS Mean ± SE LS Mean	Main Effects, p
1	b<br>Dist	Vehicle (n=6)<br>25I-NBOH (n=10)	One Way ANOVA	Vehicle: 93.140 ± 5.317<br>25I-NBOH: 108.377 ± 10.393	treatment = 0.301
	c<br>Speed	Vehicle (n=6)<br>25I-NBOH (n=10)	One Way ANOVA	Vehicle: 0.260 ± 0.0148<br>25I-NBOH: 0.301 ± 0.0289	treatment = 0.314
	d<br>Stops	Vehicle (n=6)<br>25I-NBOH (n=10)	Two Way RM ANOVA<br>Bonferroni	Vehicle: 9.778 ± 3.266<br>25I-NBOH: 19.477 ± 2.412	**treatment = 0.030**<br>time = 0.311<br>treatment x time = 0.851
	e<br>Stops	Vehicle (n=6)<br>25I-NBOH (n=10)	One Way ANOVA<br>Bonferroni	Vehicle: 117.333 ± 14.814<br>25I-NBOH: 259.182 ± 26.257	**treatment = 0.002**
2	d<br>Behav	Vehicle (n=6)<br>25I-NBOH (n=10)	Two Way RM ANOVA<br>Bonferroni	Head-twitch: 2.632 ± 0.448<br>Stop: 3.895 ± 0.448	significant interaction<br>**behavior x time <0.001**
	e<br>Behav	Vehicle (n=6)<br>25I-NBOH (n=10)	Two Way RM ANOVA<br>Bonferroni	Head-twitch: 2.632 ± 0.448<br>Stop: 3.895 ± 0.448	significant interaction<br>**behavior x time <0.001**
	g<br>Int	25I-NBOH (n=10)	Kruskal-Wallis ANOVA<br>Tukey Test	Interval 0-30: 5.707 ± 0.473<br>Interval 30-60: 9.596 ± 1.124	**time period = 0.005**
3	b<br>FFT	Baseline / 25I-NBOH<br>(n=9)	Two Way RM ANOVA<br>Bonferroni	Baseline: 47.197 ± 9.694<br>25I-NBOH 0-30: 43.537 ± 9.694<br>25I-NBOH 30-60: 106.181 ± 9.694	significant interaction<br>**treatment x frequency <0.001**
	c<br>δ	Baseline / 25I-NBOH<br>(n=9)	Kruskal-Wallis ANOVA<br>Dunn's Method	Baseline: 52.498 ± 2.936<br>25I-NBOH 0-30: 62.742 ± 7.844<br>25I-NBOH 30-60: 176.547 ± 37.829	**treatment <0.001**
	d<br>θ	Baseline / 25I-NBOH<br>(n=9)	Kruskal-Wallis ANOVA<br>Dunn's Method	Baseline: 57.905 ± 6.302<br>25I-NBOH 0-30: 65.967 ± 9.717<br>25I-NBOH 30-60: 125.107 ± 17.681	**treatment = 0.012**
	e<br>α	Baseline / 25I-NBOH<br>(n=9)	Kruskal-Wallis ANOVA<br>Dunn's Method	Baseline: 32.137 ± 2.163<br>25I-NBOH 0-30: 17.963 ± 1.748<br>25I-NBOH 30-60: 40.868 ± 9.141	**treatment = 0.009**
	i<br>Phase	25I-NBOH (n=9)	Two Way RM ANOVA<br>Bonferroni	Phase1: 19.846 ± 1.246<br>Phase2:8.615 ± 1.246	significant interaction<br>**phase x time <0.001**
	j<br>Int	25I-NBOH (n=11/9)	One Way ANOVA	Stop:HT Interval: 7.652 ± 0.532<br>P1:P2 Interval: 7.521 ± 0.365	interval = 0.841
4	d<br>Stop	Vehicle (n=6)<br>25I-NBOH (n=10)<br>Nic+25I-NBOH (n=10)	Two Way RM ANOVA<br>Bonferroni	Vehicle: 6.476 ± 0.738<br>25I-NBOH: 9.982 ± 0.684<br>Nic+25I-NBOH: 37.755 ± 0.738	significant interaction<br>**treatment x time = 0.011**
	e<br>Stop	Vehicle (n=6)<br>25I-NBOH (n=10)<br>Nic+25I-NBOH (n=10)	Kruskal-Wallis ANOVA<br>Dunn's Method	Vehicle: 112.800 ± 22.869<br>25I-NBOH: 266.491 ± 35.785<br>Nic+25I-NBOH: 1154.490 ± 160.973	**treatment <0.001**
	f<br>HT	25I-NBOH (n=10)<br>Nic+25I-NBOH (n=10)	Kruskal-Wallis ANOVA<br>Dunn's Method	25I-NBOH: 157.909 ± 26.117<br>Nic+25I-NBOH: 24.700 ± 3.320	**treatment <0.001**
5	b<br>FFT	Baseline (n=17)<br>25I-NBOH (n=9)<br>Nic+25I-NBOH (n=8)	Two Way RM ANOVA<br>Bonferroni	Baseline: 47.197 ± 8.081<br>25I-NBOH 0-30: 43.537 ± 8.081<br>Nic+25I-NBOH 0-30: 132.642 ± 8.081	significant interaction<br>**treatment x frequency <0.001**

	c<br>δ	Baseline (n=17)<br>25I-NBOH (n=9)<br>Nic+25I-NBOH (n=8)	One Way ANOVA<br>Bonferroni	Baseline: 52.498 ± 2.936<br>25I-NBOH 0-30: 62.742 ± 7.844<br>Nic+25I-NBOH 0-30: 134.992 ± 27.638	**treatment = 0.030**
	d<br>θ	Baseline (n=12)<br>25I-NBOH (n=6)<br>Nic+25I-NBOH (n=6)	One Way ANOVA<br>Bonferroni	Baseline: 57.905 ± 6.302<br>25I-NBOH 0-30: 65.967 ± 9.717<br>Nic+25I-NBOH 0-30: 168.586 ± 28.040	**treatment = 0.030**
	e<br>FFT	Baseline (n=17)<br>25I-NBOH (n=9)<br>Nic+25I-NBOH (n=8)	Two Way RM ANOVA<br>Bonferroni	Baseline: 47.197 ± 11.131<br>25I-NBOH 30-60: 106.181 ± 11.131<br>Nic+25I-NBOH 30-60: 93.580 ± 11.131	significant interaction<br>**treatment x frequency <0.001**
	f<br>δ	Baseline (n=17)<br>25I-NBOH (n=9)<br>Nic+25I-NBOH (n=8)	One Way ANOVA<br>Bonferroni	Baseline: 52.498 ± 2.936<br>25I-NBOH 30-60: 176.547 ± 37.829<br>Nic+25I-NBOH 30-60: 127.439 ± 25.724	**treatment <0.001**
	g<br>θ	Baseline (n=17)<br>25I-NBOH (n=9)<br>Nic+25I-NBOH (n=8)	One Way ANOVA<br>Bonferroni	Baseline: 57.905 ± 6.302<br>25I-NBOH 30-60: 125.107 ± 17.681<br>Nic+25I-NBOH 30-60: 130.413 ± 20.020	**treatment <0.001**
	h<br>P1	25I-NBOH (n=9)<br>Nic+25I-NBOH (n=8)	Two Way RM ANOVA<br>Bonferroni	25I-NBOH: 19.846 ± 2.119<br>Nic+25I-NBOH: 19.034 ± 2.119	**time < 0.001**<br>treatment = 0.793<br>treatment x time = 0.142
	i<br>P2	25I-NBOH (n=9)<br>Nic+25I-NBOH (n=8)	Two Way RM ANOVA<br>Bonferroni	25I-NBOH: 8.615 ± 1.050<br>Nic+25I-NBOH: 2.812 ± 1.050	significant interaction<br>**treatment x time <0.001**
S1	a<br>Dist	Vehicle (n=6)<br>25I-NBOH (n=10)	Two Way RM ANOVA<br>Bonferroni	Vehicle: 7.830 ± 0.936<br>25I-NBOH: 8.997 ± 0.725	treatment = 0.341<br>**time <0.001**<br>treatment x time = 0.465
	b<br>Dist	Vehicle (n=6)<br>25I-NBOH (n=10)	Two Way RM ANOVA<br>Bonferroni	Vehicle: 61.269 ± 6.596<br>25I-NBOH: 67.740 ± 5.109	treatment = 0.451<br>**time <0.001**<br>treatment x time = 0.881
	c<br>Speed	Vehicle (n=6)<br>25I-NBOH (n=10)	Two Way RM ANOVA<br>Bonferroni	Vehicle: 0.262 ± 0.0314<br>25I-NBOH: 0.299 ± 0.0243	treatment = 0.358<br>**time <0.001**<br>treatment x time = 0.512
	d<br>Stops	Vehicle (n=6)<br>25I-NBOH (n=10)	One Way ANOVA<br>Bonferroni	Vehicle: 112.800 ± 22.869<br>25I-NBOH: 266.491 ± 35.785	**treatment = 0.010**
S2	a<br>FFT	Baseline / 25I-NBOH<br>(n=9)	Two Way RM ANOVA<br>Bonferroni	Baseline: 9.277 ± 1.521<br>25I-NBOH 0-30: 8.315 ± 1.521<br>25I-NBOH 30-60: 17.284 ± 1.521	significant interaction<br>**treatment x frequency <0.001**
	b<br>β	Baseline / 25I-NBOH<br>(n=9)	One Way ANOVA	Baseline: 7.404 ± 0.822<br>25I-NBOH 0-30: 9.389 ± 1.222<br>25I-NBOH 30-60: 12.589 ± 2.022	treatment = 0.099
	c<br>γ	Baseline / 25I-NBOH<br>(n=9)	One Way ANOVA	Baseline: 2.358 ± 0.303<br>25I-NBOH 0-30: 2.741 ± 0.495<br>25I-NBOH 30-60: 1.980 ± 0.272	treatment = 0.457
S3	a<br>Stops	Vehicle (n=6)<br>25I-NBOH (n=10)<br>Nic+25I-NBOH (n=10)<br>Nicotine (n=4)	Two Way ANOVA<br>Bonferroni	Vehicle: 194.117 ± 84.927<br>25I-NBOH: 240.932 ± 62.722<br>Nic+25I-NBOH: 895.220 ± 65.784<br>Nicotine: 93.750 ± 104.013<br>0-30: 274.479 ± 57.329<br>30-60: 437.531 ± 57.329	significant interaction<br>**treatment x time period = 0.044**

	b<br>HT	25I-NBOH (n=10)<br>Nic+25I-NBOH (n=10)<br>Nicotine (n=4)	Kruskal-Wallis ANOVA<br>Dunn's Method	25I-NBOH: 157.909 ± 26.117<br>Nic+25I-NBOH: 24.700 ± 3.320<br>Nicotine: 1.500 ± 0.975	**treatment <0.001**
S4	a<br>FFT	Baseline (n=16)<br>Nic+25I-NBOH (n=8)	Two Way RM ANOVA<br>Bonferroni	Baseline: 47.197 ± 8.916<br>Nic+25I-NBOH 0-30: 132.642 ± 8.916<br>Nic+25I-NBOH 30-60: 93.580 ± 8.916	significant interaction<br>**treatment x frequency <0.001**
	b<br>δ	Baseline (n=16)<br>Nic+25I-NBOH (n=8)	Kruskal-Wallis ANOVA<br>Dunn's Method	Baseline: 52.498 ± 2.936<br>Nic+25I-NBOH 0-30: 134.992 ± 27.638<br>Nic+25I-NBOH 30-60: 127.439 ± 25.724	**treatment <0.001**
	c<br>θ	Baseline (n=16)<br>Nic+25I-NBOH (n=8)	Kruskal-Wallis ANOVA<br>Dunn's Method	Baseline: 57.905 ± 6.302<br>Nic+25I-NBOH 0-30: 168.586 ± 28.040<br>Nic+25I-NBOH 30-60: 130.413 ± 20.020	**treatment <0.001**
	d<br>FFT	Baseline (n=16)<br>Nic+25I-NBOH (n=8)	Two Way RM ANOVA<br>Bonferroni	Baseline: 9.277 ± 1.236<br>Nic+25I-NBOH 0-30: 19.928 ± 1.236<br>Nic+25I-NBOH 30-60: 14.823 ± 1.236	significant interaction<br>**treatment x frequency <0.001**
S5	a<br>FFT	Baseline (n=17)<br>25I-NBOH (n=9)<br>Nic+25I-NBOH (n=8)	Two Way RM ANOVA<br>Bonferroni	Baseline: 9.277 ± 1.250<br>25I-NBOH 0-30: 8.315 ± 1.250<br>Nic+25I-NBOH 0-30: 19.928 ± 1.250	significant interaction<br>**treatment x frequency <0.001**
	b<br>α	Baseline (n=17)<br>25I-NBOH (n=9)<br>Nic+25I-NBOH (n=8)	One Way ANOVA<br>Bonferroni	Baseline: 32.137 ± 2.163<br>25I-NBOH 0-30: 17.963 ± 1.748<br>Nic+25I-NBOH 0-30: 23.369 ± 4.092	**treatment = 0.002**
	c<br>β	Baseline (n=17)<br>25I-NBOH (n=9)<br>Nic+25I-NBOH (n=8)	One Way ANOVA	Baseline: 7.404 ± 0.822<br>25I-NBOH 0-30: 9.389 ± 1.222<br>Nic+25I-NBOH 0-30: 5.909 ± 0.889	treatment = 0.111
	d<br>γ	Baseline (n=17)<br>25I-NBOH (n=9)<br>Nic+25I-NBOH (n=8)	One Way ANOVA	Baseline: 2.358 ± 0.303<br>25I-NBOH 0-30: 2.741 ± 0.495<br>Nic+25I-NBOH 0-30: 1.313 ± 0.253	treatment = 0.052
	e<br>FFT	Baseline (n=17)<br>25I-NBOH (n=9)<br>Nic+25I-NBOH (n=8)	Two Way RM ANOVA<br>Bonferroni	Baseline: 9.277 ± 1.676<br>25I-NBOH 30-60: 17.284 ± 1.676<br>Nic+25I-NBOH 30-60: 14.823 ± 1.676	significant interaction<br>**treatment x frequency <0.001**
	f<br>α	Baseline (n=17)<br>25I-NBOH (n=9)<br>Nic+25I-NBOH (n=8)	One Way ANOVA	Baseline: 32.137 ± 2.163<br>25I-NBOH 30-60: 40.868 ± 9.141<br>Nic+25I-NBOH 30-60: 31.179 ± 4.335	treatment = 0.794
	g<br>β	Baseline (n=17)<br>25I-NBOH (n=9)<br>Nic+25I-NBOH (n=8)	One Way ANOVA	Baseline: 7.404 ± 0.822<br>25I-NBOH 30-60: 12.589 ± 2.022<br>Nic+25I-NBOH 30-60: 8.410 ± 1.055	treatment = 0.061
	h<br>γ	Baseline (n=17)<br>25I-NBOH (n=9)<br>Nic+25I-NBOH (n=8)	One Way ANOVA	Baseline: 2.358 ± 0.303<br>25I-NBOH 30-60: 1.980 ± 0.272<br>Nic+25I-NBOH 30-60: 1.518 ± 0.137	treatment = 0.051
